# Oroxylin A inhibits the generation of Tregs in non-small cell lung cancer

**DOI:** 10.18632/oncotarget.17218

**Published:** 2017-04-19

**Authors:** Le Shen, Lu-Lu Zhang, Hui Li, Xiao Liu, Xiao-Xuan Yu, Po Hu, Hui Hui, Qing-Long Guo, Shuai Zhang

**Affiliations:** ^1^ State Key Laboratory of Natural Medicines, Jiangsu Key Laboratory of Carcinogenesis and Intervention, School of Basic Medicine and Clinical Pharmacy, China Pharmaceutical University, Nanjing 210009, People's Republic of China; ^2^ Department of Toxicology, The Key Laboratory of Modern Toxicology of Ministry of Education, School of Public Health, Nanjing Medical University, Nanjing 211166, People's Republic of China; ^3^ Department of Thoracic Surgery, Nanjing Medical University Affiliated Cancer Hospital, Jiangsu Key Laboratory of Molecular and Translational Cancer Research, Cancer Institute of Jiangsu Province, Nanjing 210009, People's Republic of China

**Keywords:** oroxylin A, tregs, TGF-β1, NF-κB

## Abstract

Oroxylin A (OA), a naturally occurring monoflavonoid isolated from *Scutellariae radix*, has previously been reported to inhibit the proliferation of several cancer cell lines. CD4^+^CD25^+^Foxp3^+^ regulatory T cells (Tregs) play an important role in maintenance of immunologic self-tolerance. Tregs also increase in cancer and take part in suppressing antitumor immune responses. Here, we explored how OA affected the Tregs in lung cancer environment and the involved underlying mechanism. It is found that OA reversed the generation of Tregs induced by H460 lung cancer cells co-culture. Furthermore, *in vivo*, OA reduced tumor formation rate and attenuated Foxp3 expression in tumor-infiltrating lymphocytes. We also found that transforming growth factor-β1 (TGF-β1) neutralizing antibody reversed the enhancement of Treg number and expression of p-Smad3ˎ p-p38ˎp-JNKˎp-ERK1/2 in the co-culture model. Moreover, OA reduced the secretion of TGF-β1 and down-regulated the activation of NF-κB signaling in H460 cells. OA also inhibited Treg activity by a direct inhibition of the T cells' response to TGF-β1. In conclusion, our study demonstrated that OA inhibits the generation of Tregs in lung cancer environment by inhibiting the T cells' response to TGF-β1 and decreasing the secretion of TGF-β1 in lung cancer cells via NF-κB signaling.

## INTRODUCTION

Studies have shown that CD4^+^CD25^+^Foxp3^+^ regulatory T cells (Tregs) play an important role in mantaining self-tolerance and immune homeostasis. Tregs can control various immune responses by recognizing both self and nonself antigens [1, 2]. Tregs are crucial to limiting host autoimmunity, when Tregs are depleted, the mice develop autoimmunediseases [3]. However, Tregs are abundant in many cancers, such as lung cancer [4], ovarian cancer [5], liver cancer [6], lymphoma [7]. Increased Tregs in blood, tumors or lymphoid tissues of cancer patients are also associated with poor prognosis [8, 9]. Tregs that promote self-tolerance may also suppress potential responsiveness to autologus tumors in cancer patients [10].

The mechanism of tumor-infiltrating Tregs is controversial. Previous studies have shown two views: on the one hand, chemotactic cytokines secreted by tumor cells are involved in the recruitment of peripheral blood cells to the tumor tissue [11]. On the other hand, transforming growth factor-β (TGF-β) plays an important role in generation of inducible Tregs (iTregs) from CD4^+^CD25^−^ cells [12]. Tregs are produced in thymus as a mature T-cell subpopulation, Tregs can be induced from CD4^+^CD25^−^ naïve T (nT) cells under certain conditions [13]. For example, in the presence of TGF-β, CD4^+^CD25^−^ nT cells convert into Tregs involving induction of Foxp3 expression [14]. TGF-β is a member of the TGF superfamily. Three highly homologous isoforms of TGF-β exist in humans: TGF-β1, TGF-β2 and TGF-β3 [15], TGF-β1 plays the most important role of three. TGF-β1 secreted by cancer cells also can induce CD4^+^ nT cells to transform into Tregs, and knockdown of TGF-β1 reversed the process [16].

Recently, many strategies have been reported to suppress Tregs generation for promoting immunity in cancer. A p300 inhibitor can diminish Tregs number and inhibit tumor growth in immunocompetent mice without impairing T effector cell responses [17]. In addition, COX-2 inhibition significantly reduced the Tregs population by 60% at the tumor site in mice and enhanced antitumor responses [18]. Thus, drug is promising in targeting Tregs for cancer immunotherapy. However, little research about natural products was reported. In our study, we report that Oroxylin A (OA), which is a flavonoid isolated from *Scutellariae radix*, inhibits the Tregs generation in non-small cell lung cancel. OA was widely used for anti-inflammation, anticancer, antiviral and antibacterial infections [19]. Effective dose of OA shows no significant damage to normal cells and tissues, which is the advantage different from general chemotherapy drugs.

In this study, we sought to determine whether OA contributed to decreasing host antitumor immune responses by affecting Tregs in non-small cell lung cancer. We established a co-culture model and tried to investigate the influence of OA on Tregs. We suggest that the effects of OA on Tregs generation merit further investigation as a candidate for lung cancer combination therapy as a kind of immune enhancer.

## RESULTS

### OA reverses the Tregs generation induced by H460 cells co-culture

**Figure 1:**
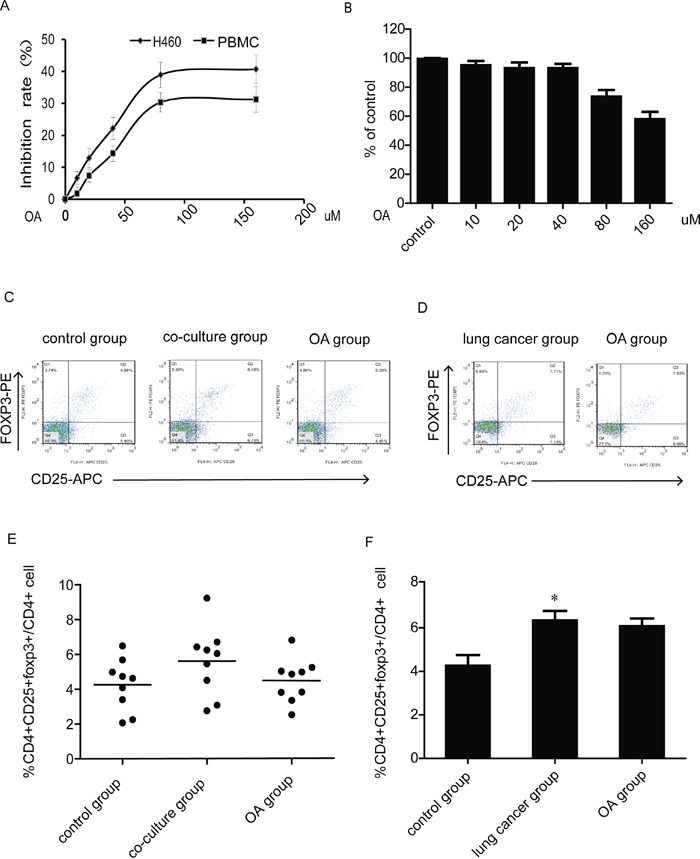
Effects of OA on Tregs generation in co-culture model **(A)** Growth inhibition of OA treatment for 24 h on PBMC was assessed by MTT assay. **(B)** Viable cells were counted using a hemocytometer after trypan blue staining to assess antigrowth effect of OA treatment for 24 h on PBMC. **(C, E)** The changes of CD4^+^CD25^+^foxp3^+^ T cells population after PBMC co-cultured with H460 lung cancer cells and treated with OA (40 μM) were detected by flow cytometry, PBMC were separated from blood donated by healthy volunteers. CD4,CD25 and foxp3-positive ratio of CD4-positive cells is shown. **(D, F)** The changes of the CD4^+^CD25^+^foxp3^+^ T cells population after PBMC treated with OA (40 μM) were detected by flow cytometry, PBMC were separated from blood donated by lung cancer patients. CD4,CD25 and foxp3-positive ratio of CD4-positive cells is shown. Each experiment was performed at least three times. Data are presented as mean ± SD. The comparisons were made relative to control group and significance of difference is indicated as *P < 0.05.

Peripheral blood mononuclear cell (PBMC) were separated from blood donated by healthy volunteers, the effects of OA on the growth of H460 cells and PBMC at various concentrations were tested by MTT assay. OA (40 μM, 24 h) shows a little growth inhibition on both cells (Figure 1A), but as shown in trypan blue staining experiment (Figure 1B), OA (40 μM, 24 h) shows no effect on PBMC viability. Then PBMC were co-cultured with H460 cells for 24 h, PBMC were harvested and analyzed for proportion of CD4^+^CD25^+^Foxp3^+^ cells via FACS analysis. The Tregs proportion was evidently increased after co-culture with H460 cells. When treated with OA in co-culture model, Tregs generation was reversed (Figure 1C, 1E). PBMC were also separated from blood donated by lung cancer patients and treated with OA (40 μM). Tregs proportion in OA- treated group showed no significant change compared with control group (Figure 1D, 1F).

### OA reduces tumor formation rate and Tregs number in C57BL/6 mice

**Figure 2:**
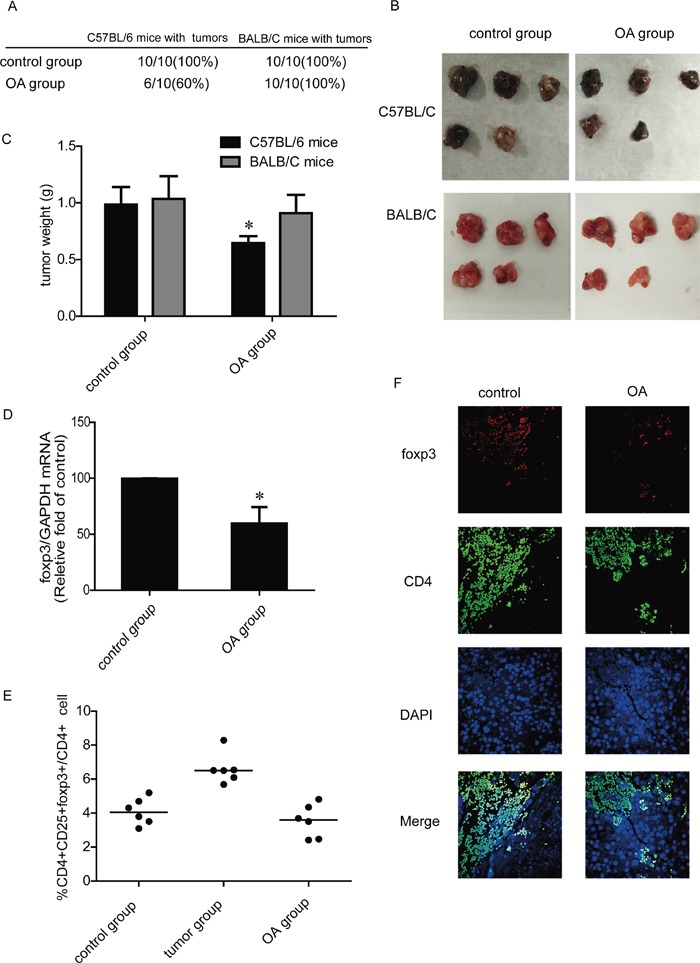
Effects of OA on Tregs generation *in vivo* **(A)** Incidence of tumors arising in C57BL/6 mice and BALB/C mice after administration of 0.9% normal saline or OA (60 mg/kg). **(B)** Macroscopic appearance of Lewis and H460 xenografts treated with 0.9% normal saline or OA (60 mg/kg). **(C)** Tumor weight of Lewis and H460 xenografts treated with 0.9% normal saline or OA (60 mg/kg). **(D)** The mRNA levels of Foxp3 in Lewis xenografts were detected by RT-PCR. **(E)** The changes of the CD4^+^CD25^+^foxp3^+^ T cells population after treated with 0.9% normal saline or OA (60 mg/kg) were detected by flow cytometry, lymphocytes were separated from mice spleen. **(F)** Immunofluorescence of paraffinembedded tissue sections from the tumors costained with CD4 and Foxp3, as well as DAPI, to visualize the nuclei (original magnification, ×1,000). Data are presented as mean ± SD. The comparisons were made relative to control group and significance of difference is indicated as *P < 0.05.

CD4^+^CD25^+^Foxp3^+^ Tregs with immunosuppressive capacity are enriched in lung cancer tumor-infiltrating lymphocytes [20]. We therefore tested the effects of OA on tumor formation and Tregs number in C57BL/6 murine lung cancer models. In one model, mice were treated with 0.9% normal saline or OA (60 mg/kg) immediately after inoculation of lung cancer cells Lewis. Palpable subcutaneous tumors arose in 10 of 10 (100%) in control group and 6 of 10 (60%) in OA group (Figure 2A). In another model, Lewis cells were inoculated subcutaneously, and then mice were uniformly divided into two groups when the tumors reached 50∼100 mm^3^. Mice were treated with 0.9% normal saline or OA (60 mg/kg) for 2 weeks. Lewis xenografts in OA- treated group had a mean tumor weight of 644±141 mg compared to saline-treated control group with a mean tumor weight of 986±342 mg; p < 0.05 (Figure 2B, 2C). We then found that OA decreased mRNA expression of Foxp3 in xenografts (Figure 2D). In addition, we tested Tregs proportion in T lymphocytes separated from spleen of mice. Results showed that Tregs proportion in lung cancer model mice increased compared with control mice, and treatment of OA also reduced the Tregs proportion significantly (Figure 2E). To visually observe tumor-infiltrating Tregs in xenografts, we detected CD4 and Foxp3 expression to identify Tregs in tissue section of tumors by double immunofluorescence stains, positive expression of Foxp3 was used to locate Tregs cells because CD25 is not specific for these cells. Results showed that OA decreased tumor-infiltrating Tregs obviously (Figure 2F).

To study the effects of OA on lung cancer without T cells involvement, immunodeficiency BALB/C mice were used as another two animal models. In one model, mice were treated with 0.9% normal saline or OA (60 mg/kg) every other day immediately after inoculation of lung cancer cells H460. We found that the number of BALB/C mice in OA- treated group exhibiting tumors (10 of 10; 100%) was the same to that of saline-treated mice (10 of 10; 100 %) (Figure 2A). In another model, when the tumors reached 50∼100 mm^3^, mice were treated with intragastric administration of 0.9% normal saline or OA (60 mg/kg) every other day for 2 weeks. Result showed that OA exerted no marked effect on tumors weight of BALB/C mice (Figure 2B, 2C).

### H460 coculture-induced Tregs generation is TGFβ-dependent and OA inhibits TGFβ-activated signaling pathway in Jurkat cells

**Figure 3:**
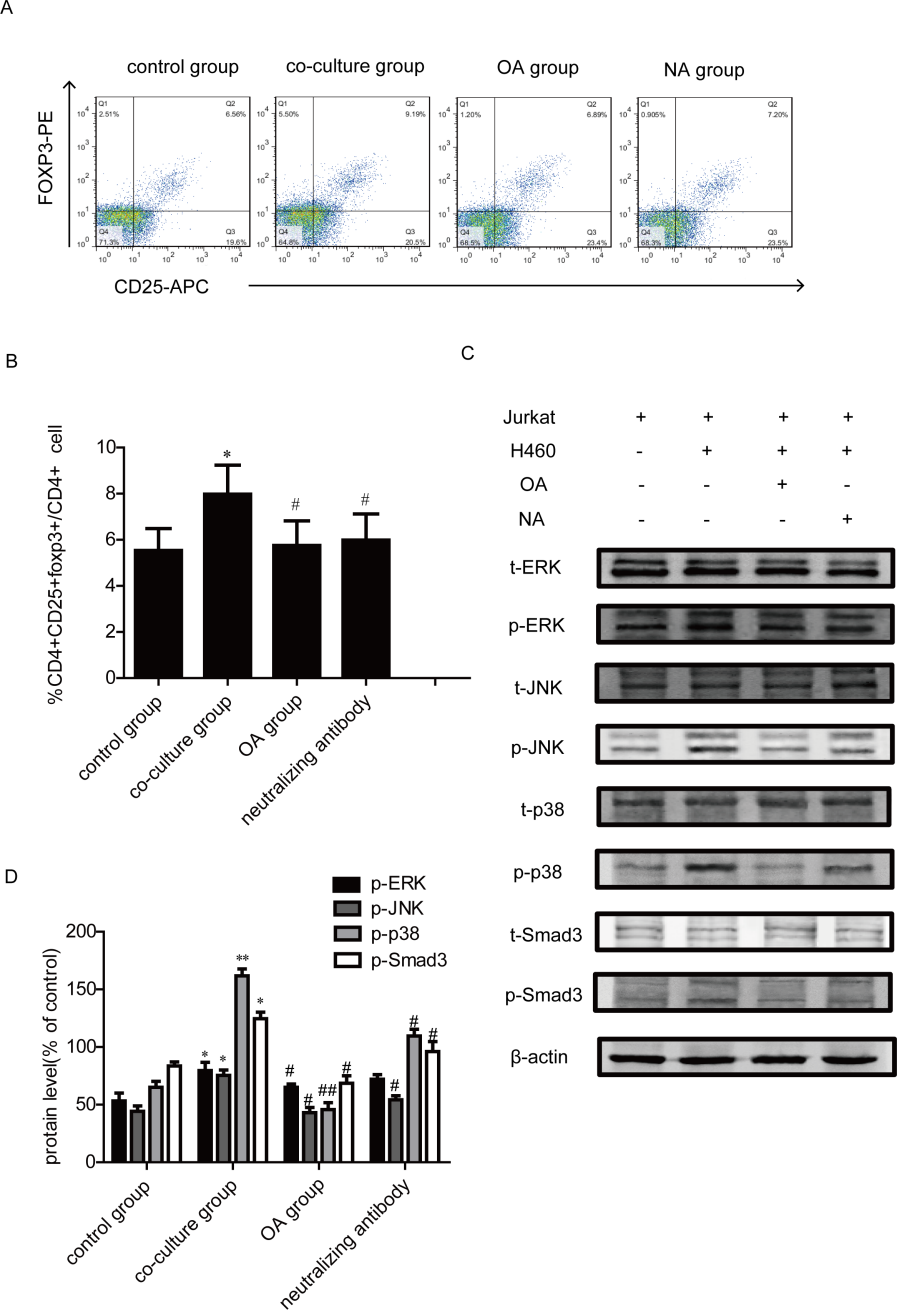
Effects of TGF-β1 neutralizing antibody on Tregs generation in co-culture model **(A, B)** The changes of the CD4^+^CD25^+^foxp3^+^ T cells population after PBMC co-cultured with H460 lung cancer cells and treated with OA (40 μM) or TGF-β1 neutralizing antibody were detected by flow cytometry. PBMC were separated from blood donated by healthy volunteers. CD4,CD25 and foxp3-positive ratio of CD4-positive cells is shown. “NA group” represents neutralizing antibody group. **(C, D)** Jurkat cells were treated with OA (40 μM) or TGF-β1 neutralizing antibody for 24 h, and TGFβ-related protein expression levels were analyzed by Western Blot. Bands were quantified, each of the protein bands was derived from different gels. Data are presented as mean ± SD, significance of difference is indicated as ^*^P < 0.05, ^**^P < 0.01, compared with control group; ^#^P < 0.05, ^##^P < 0.01, compared with co-culture group.

Lung cancer cells are known to overexpress TGF-β [4]. TGF-β has been reported to promote the conversion of CD4^+^ nT cells to Tregs by up-regulating expression of Foxp3 [21]. To explore whether co-culture with H460 cells affects Tregs by regulation of TGF-β, the TGF-β neutralizing antibody was added to the co-culture system for our study. We found that the Tregs increasement could be reversed by treatment of TGF-β1 neutralizing antibody for 24 h in co-culture group (Figure 3A, 3B). Moreover, to address if OA affected TGF-β-activated signaling pathway, OA was then used to stimulate Jurkat cells, which are co-cultured with H460 cells. Jurkat is a cell line of human T lymphocytes, it is frequently employed as T-cell model system to study immunological effects [22]. We used Jurkat cells to study the mechanisms of OA affecting Tregs. After co-culture with H460 for 24 h, the phosphorylation of Smad3, ERK, JNK and p38 were activated, which were also reversed by treatment with OA or TGF-β1 neutralizing antibody (Figure 3C, 3D).

### OA reduces the secretion of TGF-β1 in H460 cells and inhibits Tregs activity by a direct inhibition of the T cells' response to TGF-β1

**Figure 4:**
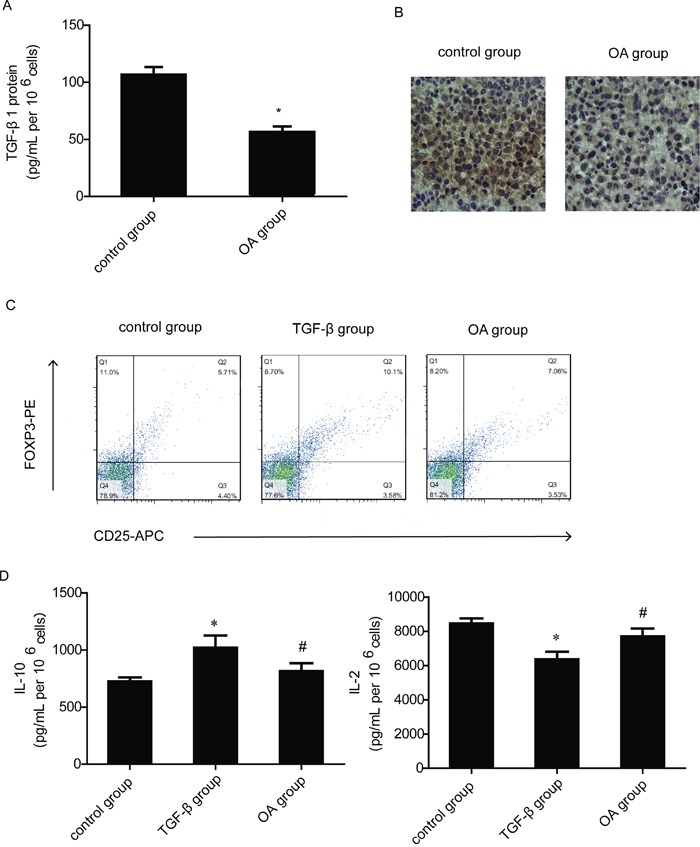
Effects of OA on TGF-β1 production and T cells' response to TGF-β1 **(A)** The level of TGF-β1 release was measured using ELISA assay from OA- treated (40 μM) H460 cell culture supernatants. **(B)** IHC analysis of H460 xenograft, obtained after subcutaneous transplantation in BALB/C mice as described in the Methods. Result shown is from one representative paraffinized specimen out of three studied. The micrographs were imaged at 200×magnifications. **(C)** The changes of CD4+CD25+foxp3+ T cells population after CD4^+^ nT cells cultured in induction model and treated with OA (40 μM) were detected by flow cytometry, CD4^+^ nT cells were separated from blood donated by healthy volunteers. CD4,CD25 and foxp3-positive ratio of CD4-positive cells is shown. **(D)** The level of IL-2 and IL-10 release was measured using ELISA assay from cell culture supernatants in Tregs induction model. Each experiment was performed at least three times. Data are presented as mean ± SD, significance of difference is indicated as *P < 0.05, compared with control group; ^#^P < 0.05, compared with co-culture group.

We then studied the effects of OA on TGF-β production. TGF-β1 plays the most important role in three isoforms of TGF-β. Enzyme-linked immunosorbent assay (ELISA) was used to determine the secretion of TGF-β1 from diluted cell-free supernatants of H460 cells. OA significantly reduced the secretion of TGF-β1 in H460 cells (Figure 4A). Morever, IHC analysis of H460 xenograft, obtained after subcutaneous transplantation in BALB/C mice as described in the Methods, shows that TGF-β1 was significantly reduced in tumors obtained from animals treated with OA (Figure 4B). In the next experiment, CD4^+^ nT cells were cultured in Tregs cell induction model with TGF-β1 and IL-2 as described in the Methods. After 72 h, the cells were analyzed by flow cytometry. TGF-β1 increased the proportion of Tregs and OA inhibited the proportion of Tregs induced by TGF-β1 significantly (Figure 4C). The culture supernatant was also analyzed for IL-2 and IL-10 secretion. We observed a significant increase in IL-10 secretion with a concomitant decrease in IL-2 following 72h of CD4^+^ T cells culture with TGF-β1, compared to the medium control groups. Treatment with OA significantly reversed TGF-β1-mediated enhancement or inhibition of IL-10 and IL-2 (Figure 4D).

### OA inhibits NF-κB signaling pathway in H460 cells

**Figure 5:**
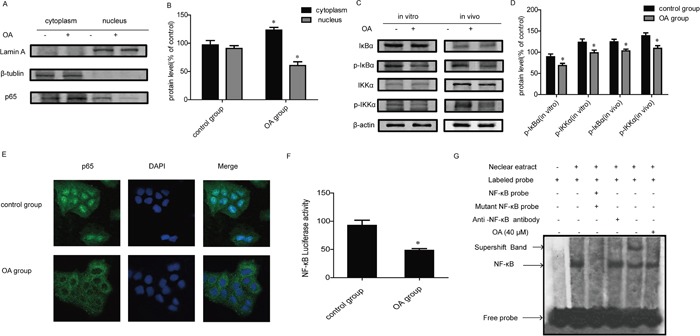
Effects of OA on NF-κB signaling pathway **(A, B)** NF-κB p65 nuclear translocation were determined by Western Blot. Lamin A and β-tublin were used as nuclear and cytoplasmic markers, respectively. Bands were quantified, each of the protein bands was derived from different gels. **(C, D)** IκBα and IKKα expression and phosphorylation were determined by Western Blot. Bands were quantified, each of the protein bands was derived from different gels. Protein *in vivo* was extracted from H460 xenograft, obtained after subcutaneous transplantation in BALB/C mice as described in the Methods. **(E)** Immunofluorescence of the effect of OA on p65 nuclear translocation (original magnification, ×1,000). **(F)** The transcriptional activities of NF-κB in H460 cells cotransfected with pNFκB-luc and pRL-TK Renilla with OA (40 μM). Luciferase activity was determined 24 h posttreatment by promega dual luciferase reporter assay system, normalized against values for the corresponding pRL-TK Renilla activity. **(G)** NF-κB DNA binding activity was detected by EMSA in H460 cells treated with OA (40 μM) for 24 h. Each experiment was performed at least three times. Data are presented as mean ± SD. The comparisons were made relative to control group and significance of difference is indicated as *P < 0.05.

P65 is a functional active subunit of NF- κB [23]. We next examined the activation and translocation of p65 in OA- treated H460 cells. Nuclei and cytoplasm protein of cells were isolated, and the amount of p65 was quantified by Western Blot. Results showed that the level of p65 protein was evidently increased in the cytoplasm and decreased in the nucleus after treatment with OA for 24 h (Figure 5A, 5B). NF-κB activity is regulated through its interaction with IκBs, the most prominent and well-studied member of which is IκBα [24]. IκBα prevents the DNA binding of NF-κB and can be phosphorylated by IKKα for triggering degradation process [25]. Then we examined whether OA regulated the phosphorylation of IκBα and IKKα. Compared with control, OA inhibited the phosphorylation of IκBα and IKKα without obvious influence on their protein expressions *in vitro* as well as *in vivo* (Figure 5C, 5D). The result of immunofluorescence shows that the level of p65 protein was evidently decreased in the nucleus after treatment with OA for 24 hours (Figure 5E). These findings suggested that OA suppressed the activation of p65 through the decreasing of p-IKKα and p-IκBα. We also measured NF-κB activation by the luciferase reporter assay. H460 cells were co-transfected with GFP and the pNF-κB-Luc plasmid, and then were treated with or without OA. A significant decrease of luciferase activity was observed in OA- treated cells compared with control group (Figure 5F). We further evaluated the effect of OA on DNA-binding activity of NF-κB by electrophoretic mobility shift assay (EMSA) assay. Results showed that OA suppressed DNA-binding activity of NF-κB in H460 cells while addition of cold NF-κB consensus oligonucleotide (100-fold) abolished them mobility shift band, demonstrating the specificity of protein–DNA interaction (Figure 5G).

## DISCUSSION

In lung cancers, tumor-infiltrating Tregs have enhanced suppressive function compared with blood or lymph node (LN) Tregs cells [26]. It has been reported that the proportion of Tregs increases in PBMC derived from lung cancer patients [27]. In the study about the effect of OA on Tregs, we simulated a lung cancer environment model *in vitro* by culturing PBMC seperated from healthy volunteers with H460 cells. As shown in Figure 1, we obtained consistent experiment results that the proportion of Tregs in PBMC from lung cancer cells was larger than in PBMC from healthy volunteers, and could be increased in the co-culture model. The treatment of OA reversed Tregs increasement induced by co-culture with H460, but not affected the Tregs proportion in PBMC derived from lung cancer patients and nomal T lymphocytes (Supplementary Figure 1A). These findings indicated that OA might inhibited Tregs generation related to lung cancer environment.

Based on the *in vitro* results, we tested the effect of OA on Tregs in established murine lung cancer models. We found that OA decreased the tumor formation rate and tumor weight at immunocompetent mice but not at immunodeficient mice (Figure 2A, 2C). This indicates the importance of a functional immune system for the full manifestation of OA– mediated antitumor responses. We next found that OA decreased Foxp3 mRNA expression in tumors significantly (Figure 2D). Expression of the transcription factor Foxp3 has been implicated as a key element for CD4^+^CD25^+^ T regulatory cell function in mice [28]. However, Foxp3 expression in humans, unlike mice, may not be specific for cells with a regulatory phenotype [29], so we tested the Foxp3 mRNA expression only in mice. In spleens, tumor-bearing mice have high level Tregs proportion compared with control mice. Similar to the results of our *in vivo* study, OA decreased the percentage of Tregs in T lymphocytes separated from spleen of tumor-bearing mice (Figure 2E). Results of double immunofluorescence stains also showed that OA decreased Tregs number in tumors obviously (Figure 2F). The results of these studies suggested that OA also inhibted the Tregs generation *in vivo*.

TGF-β is an essential cytokine for the differentiation of CD4^+^ nT cells into CD4^+^CD25^+^Foxp3^+^ Tregs cells [30]. It has been reported that lung cancer cells can produce a high level TGF-β1 that regulates antitumor immunity [31]. The activation of Smad3 by TGF-β1 stimulation and the partial overlapping role of Smad2 play a predominant role in the generation of Tregs cells [32]. We conjectured that coculture-induced Tregs generation was related to TGF-β1. Next we found that TGF-β1 neutralizing antibody inhibited the Tregs generation induced by co-culture (Figure 3A). The phosphorylation of TGF-β receptors, which consist of type I and II, triggers a downstream signaling pathway [33]. Two routes are involved in the activation of the type I receptor: the Smad-dependent canonical pathway and the non-canonical pathways [34]. non-canonical Smad-independent pathways include various branches of MAP kinase (MAPK) pathways, Rho-like GTPase signaling pathways and so on [35]. MAPK family consists of the extracellular signal regulated protein kinase (ERK), Jun amino-terminal kinase (JNK) and p38 subfamilies [36]. In our study, we found that Tregs were induced when PBMC were co-cultured with H460 cells and then the p-Smad3, p-ERK, p-JNK, p-p38 were activated. Moreover, OA also inhibited the activation of these proteins in co-culture model (Figure 3C). These findings suggested that OA regulated the Tregs generation through effects on both Smad signal and MAPK signal.

Our following study demonstrated that OA reduced the secretion of TGF-β1 cytokines *in vitro* as well as *in vivo* (Figure 4A, 4B), it also showed no effect on expression of TGF-β receptor I in Jurkat cells (Supplementary Figure 1B). Then we sought to determine whether OA could also inhibit Tregs activity by a direct inhibition of the T cells’ response to TGF-β1. We observed significant induction of Tregs activity by recombinant TGF-β1, which was inhibited by OA (Figure 4C). Moreover, the cytokine analysis further revealed an inhibition of immunesuppressive IL-10 secretion by OA, whereas the level of IL-2 was marginally enhanced (Figure 4D). IL-2 is produced primarily by actived antigen-specific CD4^+^ and CD8^+^ T cells while IL-10 is an inhibitory cytokine mediating suppression by Tregs. This indicates OA also has a certain influence on the Tregs function.

Activator protein 1 (AP1) and hypoxia are key regulators of TGF-β1 expression levels [37]. It was reported that secretion of TGF-β1 by mesenchymal stem cells (MSCs) elevated under hypoxia [38]. Recent studies also revealed that NF-κB is related to the regulation of TGF-β1 expression [39, 40]. The RelA subunit of NF-κB (NF-κB/RelA) is necessary for the inhibition of TGF-β-induced phosphorylation, nuclear translocation, and DNA binding of Smad signaling complexes [41]. Furthermore, previous study revealed that OA prevented inflammation-related tumor by inhibiting NF-κB signaling [42]. Therefore, we next asked whether the effect of OA on TGF-β1 is mediated by inhibition on NF-κB. Our results showed that OA markedly suppressed phosphorylation of IκBα and IKKα, then decreased NF-κB nuclear translocation in H460 cells (Figure 5A–5E) and inhibited DNA binding and transcriptional activity of NF-κB *in vitro* (Figure 5F, 5G).

Reports show that in the case of tumorgenesis, increased Tregs may result from induction of CD4^+^CD25^−^ cells or chemokine-driven by chemotactic cytokines [43]. Blockade of CCL22 has been demonstrated to reduce Tregs infiltration into ovarian tumors and induce tumor rejection in a murine xenograft model [44]. Here, we only tested the effect of OA on Tregs generation, further studies will be required to determine the effect of OA on chemokine-driven. In addition, the influences on Tregs function by OA also need more reasearch.

**Figure 6:**
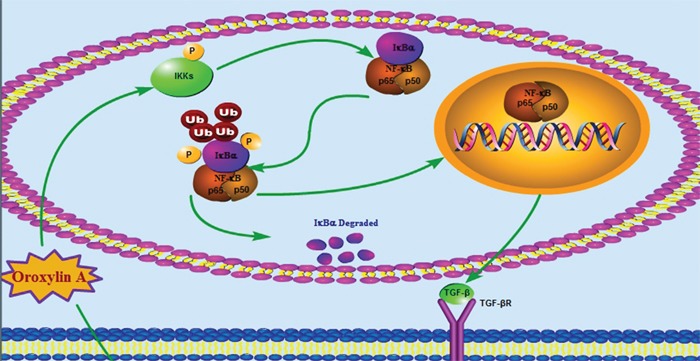
Schematic representation of the molecular mechanisms Schematic representation of the molecular mechanisms proposed in the reversal effect of OA in Tregs generation within H460 co-culture model.

In summary, our current study provided the evidence that OA inhibits the TGF-β1 secretion of lung cancer cells via suppressing NF-κB signaling so as to decrease the generation of Tregs in lung cancer environment. OA also inhibits Tregs activity by a direct inhibition of the T cells' response to TGF-β1 (Figure 6). This is the first documentation that tumor-induced immune suppression can be reversed by OA leading to the restoration of antitumor responses. This may provide an opportunity for developing a novel adjuvant therapeutic strategy for lung cancer, combining OA with immunotherapy and chemo/radiotherapeutic regimen, which could potentially improve the disease outcome.

## MATERIALS AND METHODS

### Reagents and antibodies

Oroxylin A (OA) was isolated from *Scutellariae radix* according to the protocols reported previously [19]. OA was dissolved in dimethyl sulfoxide (DMSO) as a stock solution, stored at −20°C, and diluted with medium before experiment. The final DMSO concentration did not exceed 0.1% in all experiments. 3-(4,5-dimethylthiazol-2-yl)-2,5-diphenyltetrazolium bromide (MTT), diamidinophenylindole (DAPI) were purchased from Sigma-Aldrich (St. Louis, MO). FITC anti-human CD4 (Cat. No.11-0048; mouse IgG, K) antibodies, PE anti-human Foxp3 (Cat. No.12-4777; mouse IgG, K), APC anti-human CD25 (Cat. No.17-0259; mouse IgG, K) antibodies and mouse regulatory T cell staining kit (Cat. No.88-8111; rat lgG) were obtained from eBioscience (San Diego, CA). TGF-β1 neutralization antibody were obtained from R&D Systems (Inc, Minneapolis, MN 55413 USA). Primary antibodies against β-actin, β3-tublin, IκBα, JNK, p-p38(Thr180/Tyr182), p-JNK(G-7), NF-κB(p65)(c-20), anti-mouse CD4, anti-mouse Foxp3 were obtained from Santa Cruz Biotechnology (Santa Cruz, CA). Primary antibodies against IKKα, Lamin A, p38, Smad3(S2), p-IKKα(T23) and p-Smad3(S425) were products from Bioworld (OH, USA). Primary antibodies against ERK1/2, p-IκBα(Ser32) and p-ERK1/2(Thr177/Thr160) were obtained from Cell Signaling Technology (Danvers, MA). TGF-β1 Polyclonal Antibody were products from Abclonal (Cambridge, MA, USA). FITC-conjugated anti-rabbit IgG antibody for CD4 (1:500), the Alexa Fluor^®^ 594 donkey anti-mouse IgG for Foxp3 and Alexa Fluor® 488 donkey anti-rabbit IgG for p65 (1:500) were obtained from life technologies (Eugene, OR, USA). TGF-β1 and IL-2 recombinant were obtained from PeproTech (Rocky Hill, NJ).

### Cell culture

The H460ˎ Jurkat and Lewis cell lines were purchased from the cell bank of Shanghai Institute of Biochemistry and Cell Biology, Shanghai Institutes for Biological Sciences, Chinese Academy of Sciences, Shanghai, China. Cell growth and viability was measured by MTT assay and trypan blue staining. Peripheral blood mononuclear cell (PBMC) from lung cancer patients (Jangsu Cancer Hospital, Nanjing, China) and healthy donations were collected using lymphocyte-monocyte separation medium (Jingmei, Nanjing, China). Then PBMC (1 × 10^6^ cells/ well) were co-cultured with H460 cells (5 × 10^5^ cells/ well) in a 6-well plate for 24 h. CD4^+^ nT cells were selected by using Naïve CD4^+^ T Cell Isolation Kit II (Miltenyl, 130-094-131). Human samples involved in our study were donated following written informed consent using documentation approved by the Research Ethics Committee of Jangsu Cancer Hospital. Lymphocytes were obtained from mice spleens as described [45].

### *In vitro* Tregs cell induction model

Briefly, CD4^+^ nT cells were cultured (1 × 10^6^ cells/ well) in a 6-well plate with anti-CD2/CD3/CD28-loaded MACS microbeads (Miltenyi Biotech, Auburn, CA) and low-dose IL-2 (50 IU/ml) in serum-free X-vivo medium (Lonza, Walkersville, MD). TGF-β1 (20 ng/ml) was also added in the model.

### Flow Cytometry

Cells were stained according to the manufacture's staining protocol. Flow cytometry was immediately performed on a FACS-Calibur instrument (BD Biosciences). Multiparametric analysis was carried out to detect simultaneous expression of different surface molecules on cells. In all determinations of cell surface antigen expression, gates were set to exclude dead or necrotic cells by forward/side scatter light parameters. A minimum of 10,000 events was acquired for each analysis. Results are expressed as percentage of CD4-positive cells co-expressing CD25 and Foxp3.

### Transplantation of lung cancer cells into mice

8×10^5^ Lewis and 3×10^6^ H460 tumor cells were injected s.c. in the right suprascapular area of C57BL/6 mice and BALB/C mice separately. Mice were divided into two models. In one model, mice were treated with intragastric administration of 0.9% normal saline or OA (60mg/kg) every other day until palpable tumors formed. In another model, when the tumors reached 50∼100 mm^3^, mice were treated with the same OA (60 mg/kg) every other day for 2 weeks.

### Quantitative Real-Time RT-PCR

Total RNA was extracted by the TriPureSolution (Takara Bio, Inc., Otsu, Shiga, Japan) after OA exposure, and reverse transcription was carried out by Primescript reverse transcriptase (TakaraBio, Inc.) following the manufacture's instructions. Quantitative real-time PCR for indicated genes was performed using SYBR green qPCR kit (TakaraBio, Inc.) by a fluorescent temperature cycler (real-time PCR system, Eppendorf, Hamburg, Germany). The sequences of PCR primers were listed as follows: Foxp3: 5′-CAT TTG CCA GCA GTG GGT AG-3′, 5′-CAT TTG CCA GCA GTG GGT AG-3′, with the annealing temperature of 55°C; GAPDH: 5′-ATG AGC CCC AGC CTT CTC CAT-3′ (forward), 5′-GGT CGG AGT CAA CGG ATT TG-3′ (reverse), with the annealing temperature of 55°C. mRNA level was derived from the standard curve and was expressed as the relative change after normalized versus GAPDH.

### Immunofluorescence and confocal fluorescence microscopy

Briefly, 2-mm paraffinembedded tissue sections from the tumors were processed through antigen retrieval buffer, permeabilized with Triton X-100 for 30 min, blocking of non-specific interaction with 3% BSA for 4 h, followed by washing and reaction with primary antibodies. The primary antibodies included anti-mouse CD4 (1:400), anti-mouse Foxp3 (1:200) (Santa Cruz, CA, USA). After rinsing, sections were incubated with secondary antibodies. Firstly, a FITC-conjugated anti-rabbit IgG antibody for CD4 was incubated with the tissue sample for 1 h at room temperature. Then, the Alexa Fluor^®^ 594 donkey anti-mouse IgG for Foxp3, was incubated with the sample for 1 h at room temperature. And then the coverslips were stained with DAPI for 30 min. The images were captured with an Olympus FV1000 confocal microscope. The effect of OA on p65 nuclear translocation was also performed as described above.

### Western blot analysis

Jurkat cells were co-cultured with H460 cells and treated with OA (40 μM) for 24 h. Then Jurkat cells were collected and lysed in lysis buffer (100 mM Tris–Cl, pH 6.8, 4% (m/v) SDS, 20% (v/v) glycerol, 200 mM β-mercaptoethanol, 1 mM PMSF, and 1 g/ml aprotinin) for 1 h on ice. Lysates were centrifuged at 12,000 × g for 30 min at 4°C. In another experiment, after treatment with 40 μM OA for 24 h, nuclear and cytoplasmic protein of H460 cells were extracted using Nuclear and Cytoplasmic Protein Extraction Kit according to the manufacturer's protocol. The concentration of proteins was detected using the BCA assay with a Varioskan multimode microplate spectrophotometer (Thermo, Waltham, MA, USA) at 562 nm. Subsequent steps were performed as described [46].

### Cytokine quantification by enzyme-linked immunosorbent assay (ELISA)

TGF-β, IL-2, IL-10 secretion in cell supernatants was measured by ELISA according to the manufacturer's instructions (ELISA Kit, BOSTER). The experiments were repeated three times, medium without serum was used. Levels of cytokines were expressed in pg/ml.

### Immunohistochemistry(IHC)

IHC was performed as described [47].

### Electrophoretic mobility shift assay (EMSA) of NF-κB

EMSA was performed as described [47].

### Transient transfection and luciferase assay

H460 cells were seeded in 6-well plate cultured for 12 h and then transfected with 1mg NF-κB -TA-luc (Beyotime, Nan-tong, China) and 0.05 mg pRL-TK Renilla (Beyotime) with 10 mL Lipofectamine 2000 and incubated for 24 h at 37°C with OA. Then cells, lysed by Promega passive lysis buffer, were assayed by using Promega dual luciferase (Firefly luciferase/Renilla luciferase) kit. Luciferase intensity detected with a Luminoskan Ascent (Thermo Fisher Scientific, Inc., Waltham, MA).

### Statistical analysis

Data were expressed as means SD and statistically compared by one-way ANOVA.*P<0.05, ^#^P<0.05 was taken as statistically significant and **P<0.01, ^##^P<0.01 was considered as dramatically significant. All the results were from at least three independent experiments performed in a parallel manner.

## SUPPLEMENTARY FIGURE



## Abbreviations

OA: Oroxylin A
Tregs: regulatory T cells
TGF-β1: growth factor-β1
nT cells: naïve T cells
MTT: 3-(4,5-dimethylthiazol-2-yl)-2,5-diphenyltetrazolium bromide
DAPI: diamidinophenylindole
iTregs: inducible Tregs
DMSO: dimethyl sulfoxide
PBMC: Peripheral blood mononuclear cell
ELISA: Cytokine Quantification by Enzyme-Linked Immunosorbent Assay
IHC: Immunohistochemistry
EMSA: Electrophoretic mobility shift assay
