# Supplementary material for: Oroxylin A inhibits the generation of Tregs in non-small cell lung cancer
Source: Oncotarget. 2017 Apr 19;8(30):49395–408. doi: 10.18632/oncotarget.17218 (PMC5564777; doi:10.18632/oncotarget.17218)
Supplement: Supplementary file 1 [file oncotarget-08-49395-s001.pdf]

Oroxylin A inhibits the generation of Tregs in non-small cell lung cancer

SUPPLEMENTARY MATERIAL

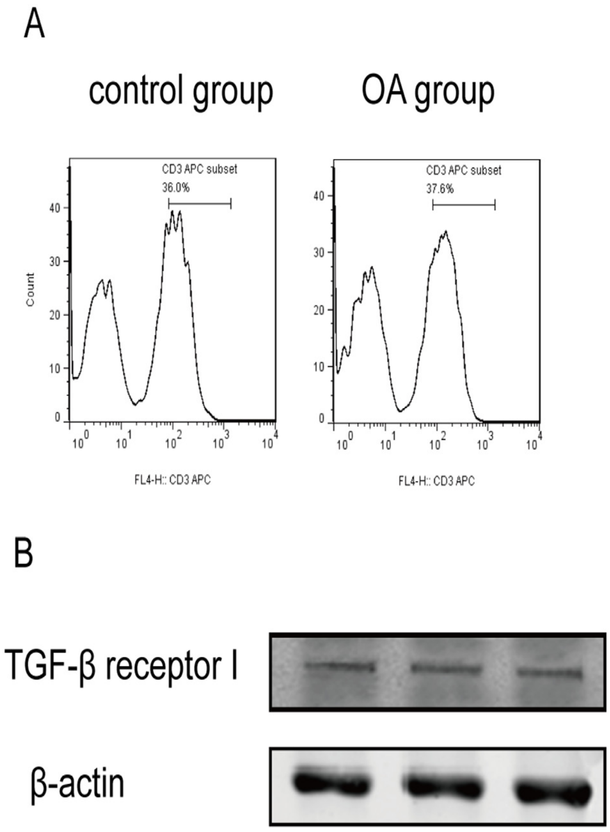

Supplementary Figure 1: (A) The changes of $CD3^+$ T cells population after PBMC treated with OA (40 μM) were detected by flow cytometry. PBMC were separated from blood donated by healthy volunteers. **(B)** Jurkat cells were treated with OA (40 μM) for 24 h, and TGF-β receptor I protein expression levels were analyzed by Western Blot.
